# Baculovirus as an efficient vector for gene delivery into mosquitoes

**DOI:** 10.1038/s41598-018-35463-8

**Published:** 2018-12-12

**Authors:** Nenavath Gopal Naik, Yu-Wen Lo, Tzong-Yuan Wu, Chang-Chi Lin, Szu-Cheng Kuo, Yu-Chan Chao

**Affiliations:** 1 Institute of Molecular Biology, Academia Sinica, No. 128, Sec. 2, Academia Road, Nankang, Taipei, 115 Taiwan Republic of China; 2 Department of Bioscience Technology, Chung Yuan Christian University, Chungli, 320 Taiwan Republic of China; 3 Department of Chemistry, Chung Yuan Christian University, Chungli, 320 Taiwan Republic of China; 4 Institute of Preventive Medicine, National Defense Medical Center, Taipei, 114 Taiwan Republic of China; 5 Department and Graduate Institute of Microbiology and Immunology, National Defense Medical Center, Taipei, 114 Taiwan Republic of China; 6 Department of Plant Pathology and Microbiology, College of Bioresources and Agriculture, National Taiwan University, Taipei, 106 Taiwan Republic of China; 7 Department of Life Sciences, College of Life Sciences, National Chung Hsing University, Taichung, 402 Taiwan Republic of China

## Abstract

Efficient gene delivery technologies play an essential role in the gene functional analyses that are necessary for basic and applied researches. Mosquitoes are ubiquitous insects, responsible for transmitting many deadly arboviruses causing millions of human deaths every year. The lack of efficient and flexible gene delivery strategies in mosquitoes are among the major hurdles for the study of mosquito biology and mosquito-pathogen interactions. We found that *Autographa californica* multiple nucleopolyhedrovirus (AcMNPV), the type baculovirus species, can efficiently transduce mosquito cells without viral propagation, allowing high level gene expression upon inducement by suitable promoters without obvious negative effects on cell propagation and viability. AcMNPV transduces into several mosquito cell types, efficiently than in commonly used mammalian cell lines and classical plasmid DNA transfection approaches. We demonstrated the application of this system by expressing influenza virus neuraminidase (NA) into mosquito hosts. Moreover, AcMNPV can transduce both larvae and adults of essentially all blood-sucking mosquito genera, resulting in bright fluorescence in insect bodies with little or no tissue barriers. Our experiments establish baculovirus as a convenient and powerful gene delivery vector *in vitro* and *in vivo* that will greatly benefit research into mosquito gene regulation, development and the study of mosquito-borne viruses.

## Introduction

Mosquitoes are primary vectors for the transmission of many human diseases such as chikungunya (CHIKV), dengue (DENV), filarial, malaria, yellow fever, and the recent Zika virus (ZIKV) outbreaks, all of which continue to pose public health risks and contribute to significant global economic losses^1,2^. *Aedes*, *Culex*, and *Anopheles* mosquito species are the most effective transmitters of deadly viruses and parasites to humans^3^. According to World Health Organization’s (WHO) latest report (April, 2017), malaria alone caused 429,000 deaths worldwide in 2015 and at least one million human deaths every year caused by the mosquito-borne diseases have been reported^4^. Despite continuous study of mosquito gene regulation and efforts to prevent mosquito-borne diseases, lack of efficient and flexible gene delivery approaches hinder investigations into virus/host interactions and mosquito biology. An efficient gene delivery system across different mosquito species into cells, larvae, and different organs of adults would obviously be an indispensable tool for such studies and have many other crucial applications in biological research.

Normally, germ line transformation technique is used to construct the stable transgenic mosquito lines to study the biological function of desired genes in the mosquitoes. However, this is a time-consuming technique and has been successful only in few mosquito species^5,6^. Classical *in vitro* plasmid transfection is a faster approach for expressing target genes, but it is associated with lower efficiency and reagent toxicity issues. Infections by viral vectors have emerged as the dominant method of choice to deliver genes in gene regulation studies. Mosquito densovirus (MDV)-mediated gene delivery has recently been developed^7^. However, MDVs are replication-competent and species-specific^8–10^, and a further drawback is the packaging limitations of DNA cargo size in MDV genomes^7^. Therefore, a better strategy for gene transfer in mosquitoes is greatly needed.

Baculovirus is a versatile tool for agricultural and biotechnological applications. The baculovirus expression vector system (BEVs) derived from this virus is popular for the production of engineered proteins^11^. This system can produce proteins with high yield and proper post-translational modifications that are suitable for various applications^12^. *Autographa californica* multiple nucleopolyhedrovirus (AcMNPV) is the type baculovirus species, which infects only lepidopteran insects and cell lines. This virus has a double-stranded, closed-circular DNA genome of 134 kb with a coding capacity of over 154 polypeptides^13^. In 1995, AcMNPV was found possible to transfer genes into mammalian cells and efficiently expressed by a promoter functional in the target cells^6,14^. It has since been successfully exploited in gene transfer applications for many mammalian cell lines, primary cells, progenitor cells, induced pluripotent (iPS) and stem cells, and is well-known as BacMam system^15,16^. Prior to these discoveries in the mammalian system, one study reported that AcMNPV may replicate at very low levels in *Aedes aegypti* mosquito cell lines, which could only be detected by a very sensitive radioisotope labeling of the viral genome^17^. However, no any further studies on AcMNPV in mosquitoes have been reported. Another study showed that *Culex nigripalpus* nucleopolyhedrovirus (CuniNPV) belongs to delta baculovirus is a pathogen of *Culex* mosquitoes, which are vectors of West Nile virus and other forms of encephalitis^18^. However, CuniNPV could only infect the genus *Culex* and it is not well characterized compared to AcMNPV. Also, CuniNPV is an infectious virus, and thus not suitable for the purposes of gene transfer to study gene regulations and/or the developments of the cells, larvae, and adults without significantly affecting the normal physiology of the mosquitoes. Unlike MDVs, the transgene capacity of AcMNPV is very large, probably extending beyond 100 kbp^19^. Baculovirus transduction into mammalian cells has been widely used to express genes driven by the mammalian promoters^14,20^. Since baculovirus is not a mammalian pathogen, it has been successfully adapted into a safe vector for gene delivery into mammalian cells, both *in vitro* and *in vivo*^21^. Baculovirus has also served as an efficient vehicle for gene transfer into *Drosophila* S2 cells, but not into larvae or adults^22^.

Here, we report the development of a baculovirus vector system for efficient gene delivery into mosquito (BacMos). This is a simple and efficient tool yet for introducing foreign genes into the cells, larvae, and adults of all tested mosquito genus that are vectors of important viral diseases. Several lines of evidence suggest that baculovirus do not replicate but efficiently transduce mosquito C6/36 cells. We first examined and compared the strength of various baculovirus-incorporated promoters in C6/36 cells, and further showed strong baculovirus-mediated foreign gene expression in larvae and in almost all adult mosquito tissues without an obvious tissue barrier. This BacMos system combines simplicity and flexibility, and serves as an alternative to existing inefficient or tedious approaches. Therefore, we believe BacMos has the potential to become a powerful tool for gene regulation analysis, which may significantly assist in advancing studies of mosquito biology and pathogen interactions.

## Results

### Baculovirus transduction into mosquito C6/36 cells

**Figure 1:**
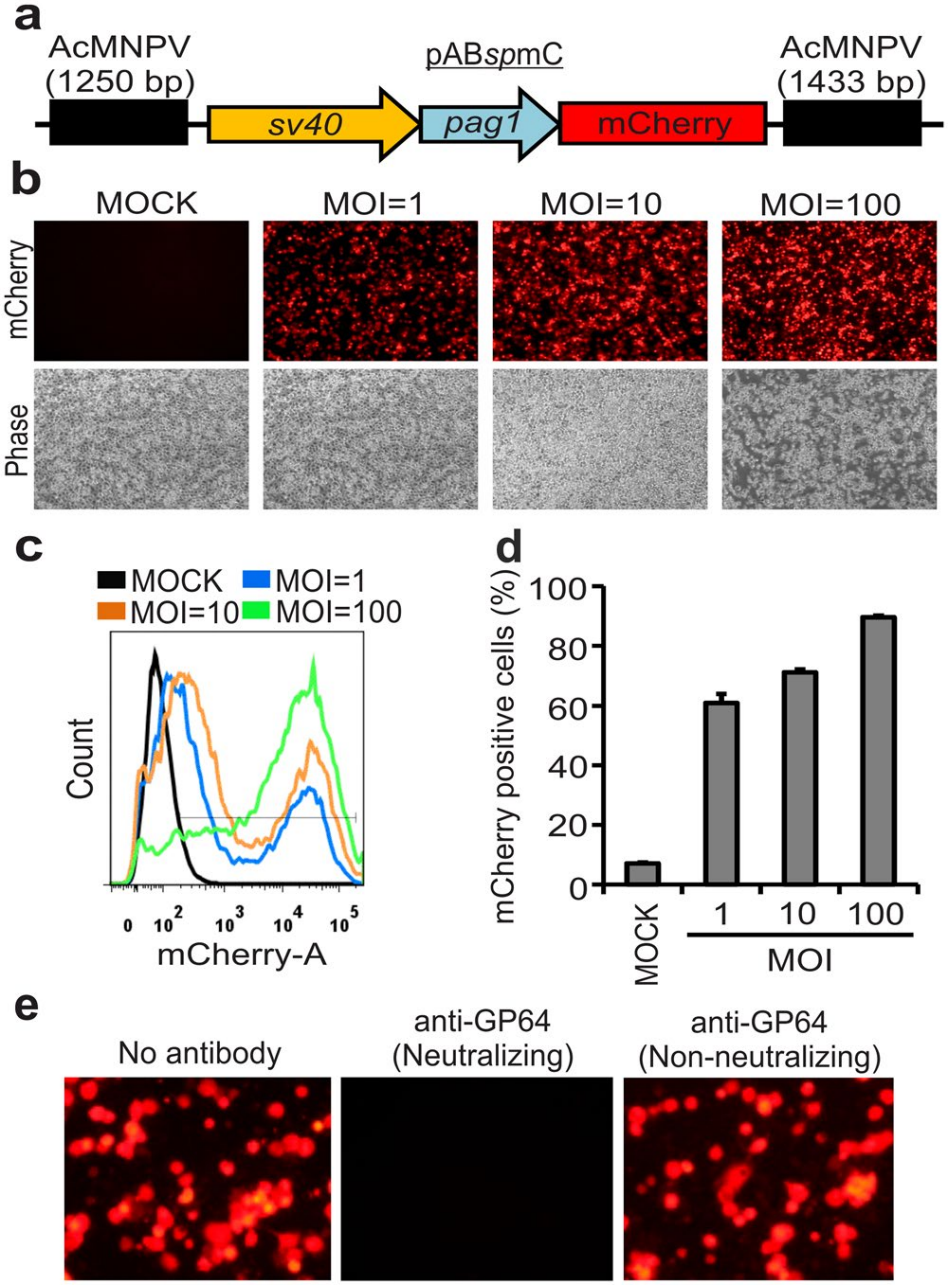
Baculovirus transduction into mosquito C6/36 cells. (**a**) Schematic representation of a composite expression vector for generating recombinant baculovirus. The composite transfer vector contains the *sv40* and *pag1* promoters, which are designed to express mCherry in mammalian and insect cells, respectively. (**b**) Dose-dependent entry of baculovirus. C6/36 cells were transduced with different concentrations (MOI = 1, 10, and 100) of recombinant vAB*sp*mC baculovirus. (**c**) Gating of mCherry-positive cells by flow cytometry, shown the efficiency of baculovirus transduction using different MOIs. (**d**) Quantification of mCherry-positive cells to indicate transduction efficiency. (**e**) GP64-mediated entry of baculovirus. C6/36 cells were treated with a baculovirus-antibody mixture (neutralizing or non-neutralizing antibodies against GP64 protein). The mCherry fluorescence images were taken at 48 h post-transduction in both panel’s (b and e). Data represent the average ± SD (standard deviation) of three biological replicates (*n* = 3).

AcMNPV is currently the most commonly used baculovirus for foreign gene expression. To examine if AcMNPV can enter into mosquito host C6/36 cells and deliver an effective gene expression cassette, we utilized recombinant baculovirus (vAB*sp*mC) containing mCherry under the control of *Heliothis zea* nudivirus 1 (*Hz*NV-1) virus early gene *pag1* promoter^23^ and *sv40* promoter^24^ (dual-promoter) that was originally designed for mammalian and insect cell systems respectively (Fig. 1a). We transduced C6/36 cells with vAB*sp*mC at MOI = 1, 10 and 100 and found mCherry fluorescence as early as 12 h (data not shown), and significant dose-dependent fluorescence intensity was detected at 48 h post-transduction (Fig. 1b). Transduced cells were then collected and analyzed by flow cytometry to determine transduction efficiency. Gating of mCherry-positive cells and quantification revealed that transduction efficiencies of 60–65% could be routinely achieved at MOI = 1, and efficiency increased to 95–100% at MOI = 100 (Fig. 1c,d). We think that mCherry could have been driven by the *pag1* promoter rather than the *sv40* promoter because thus far there is no evidence that the *sv40* promoter is functional in mosquito hosts. It is well known that baculovirus enters insect or mammalian cells through surface GP64 proteins^25^. To assess GP64-mediated cell entry, baculovirus was treated with neutralizing or non-neutralizing GP64 antibodies before being transduced into C6/36 cells. We found that baculovirus treated with GP64-neutralizing antibody did not show mCherry fluorescence at 48 h post-transduction compared to a negative control (Fig. 1e), indicating that baculovirus indeed enters mosquito hosts through the GP64 surface protein. Together, these results suggest that baculovirus can successfully enter and drive transgene expression in mosquito C6/36 cells.

### Analysis of cellular proliferation and cell viability in response to baculovirus transduction

**Figure 2:**
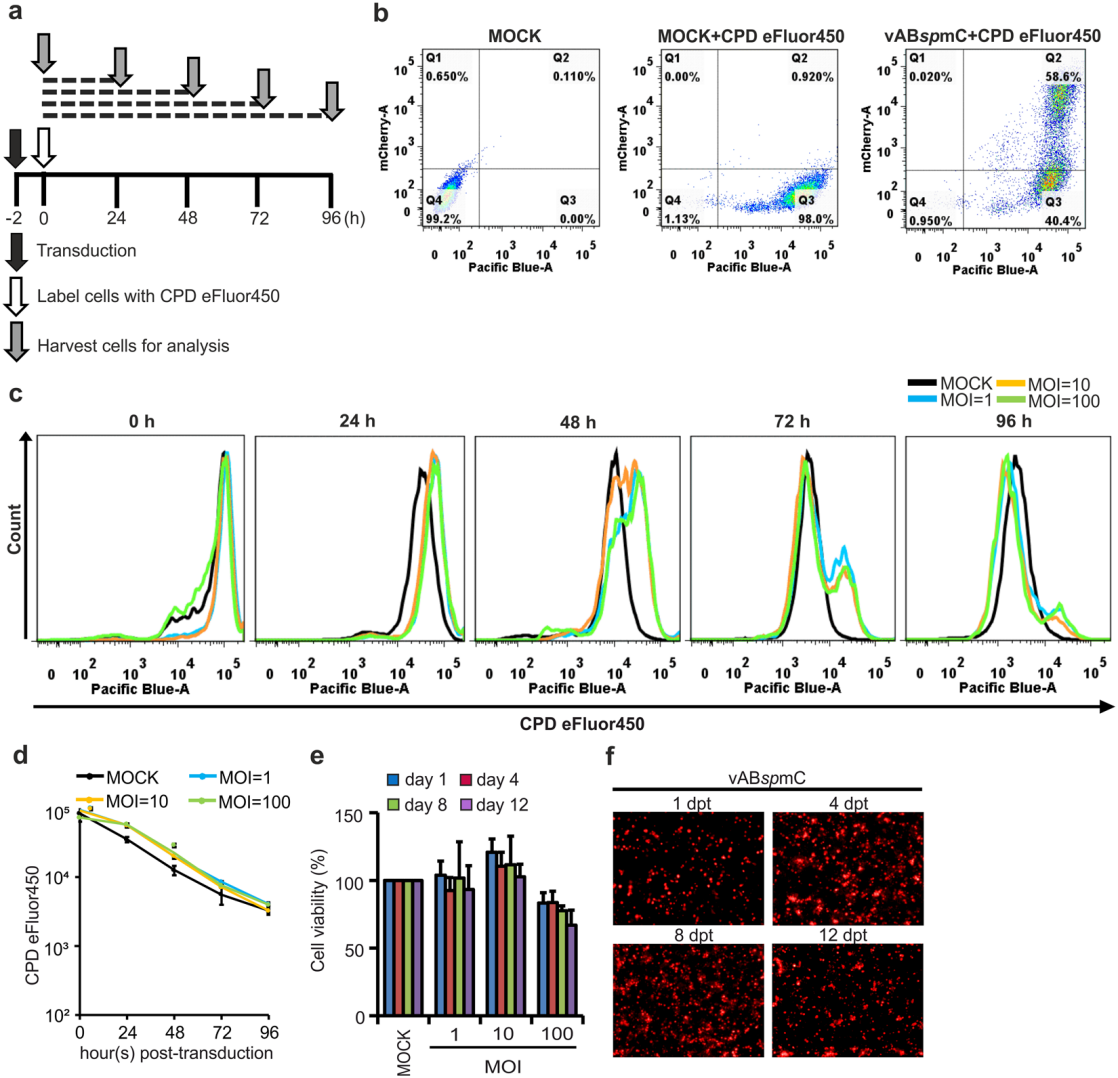
Effect of baculovirus transduction on cellular proliferation and toxicity. (**a**) Schematic diagram showing the experimental design. (**b**) Flow cytometry analysis of cells labeled with CPD eFluor450 dye. Representative dot plots of mock, mock + CPD eFluor450 and vAB*sp*mC + CPD eFluor450 (MOI = 1) for 24 h were shown as an example for gating of populations. (**c**) Representative histograms of CPD eFluor450 labeling intensity in baculovirus transduced cells (Q2 population only) and MOCK cell (Q3 population only) at the indicated time points. (**d**) Analysis of mean CPD eFluor450 labeling intensity in mock and baculovirus transduced cells with different MOIs at various time points. (**e**) Cell viability. Toxicity of baculovirus transduction into C6/36 cells at varying dosages, measured every 4 days by AlamarBlue cell viability assay. (**f**) Persistent baculovirus-mediated gene expression. C6/36 cells were transduced with baculovirus at MOI = 1 and expression of fluorescent protein was analyzed over several days. The mCherry fluorescence images at various time-points were taken by fluorescence microscopy. Data shown represent the averages of three biological replicates, with error bars showing standard deviations.

In general, the baculovirus undergoes a lytic cycle after virus infection in lepidopteran cells such as Sf9, Sf21, and High Five cells. This cycle significantly affects transcription and translation of the host genes^26,27^. To investigate whether C6/36 cells undergo the same lytic pathway and suffer the same cell proliferation effects in response to baculovirus, cell division was monitored by cell proliferation dye (CPD) eFluor450 dilution^28^. Mosquito C6/36 cells were exposed to the vAB*sp*mC virus at various MOIs, labeled with 10 µM CPD eFluor450 and cultured for up to 4 days (Fig. 2a). The cells were harvested at every 24 h interval and subjected to flow cytometry analysis to measure the dye intensities in the transduced cells (Q2 population) and mock cells (Q3 population) (Fig. 2b). We found baculovirus transduced cells showed slightly higher dye intensities at 24 and 48 h points but there is a gradual shift in the peak towards the left at later time-points reaching comparable dye intensities to mock at 96 h at all the MOIs (Fig. 2c). This data suggests that baculovirus does not significantly affect the kinetics of C6/36 cell division although there was slightly delayed in the early time points (Fig. 2d). Cell viability was analyzed at 4-day intervals by adding 10% v/v AlamarBlue to determine cell metabolic activity. There were no apparent toxicities in C6/36 cells at MOI = 1 and MOI = 10 for all exposure timeframes, but there was a mild negative effect for MOI = 100 at 12 days post-transduction (Fig. 2e). We also examined whether C6/36 cells support persistent gene expression and simultaneously analyzed the toxicity of baculovirus to C6/36 cells for up to 12 days. The mCherry expression increased as a function of time up to 8 days post baculovirus transduction before reaching saturation (Fig. 2f). Thus, BacMos expression system does not involve the baculovirus lytic cycle, no significant effect on cell proliferation kinetics and C6/36 cells can sustain baculovirus transduction for long periods of time.

### Replication analysis of baculovirus in mosquito C6/36 cells

**Figure 3:**
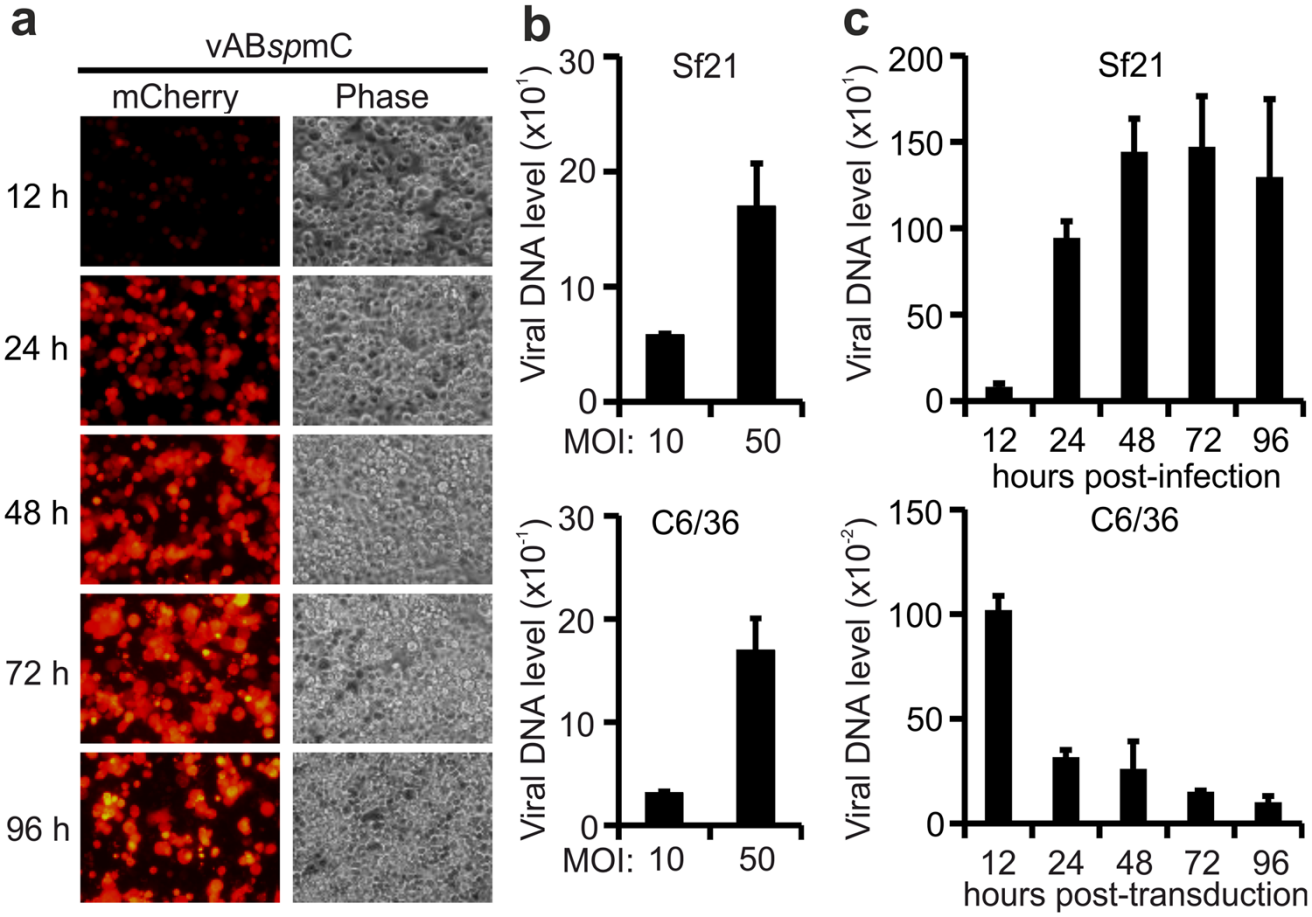
Replication analysis of baculovirus in C6/36 cells. (**a**) mCherry fluorescence images of baculovirus transduction. C6/36 cells were transduced with vAB*sp*mC at MOI = 10 and mCherry fluorescence images were captured by fluorescence microscopy at various time-points as shown. (**b**) Baculovirus entry efficiency. Baculovirus was transduced into C6/36 cells or infected with MOI = 10 and MOI = 50 for 2 h into Sf21 cells before relative entry efficiencies were quantified by qPCR. (**c**) Replication efficiency of baculovirus. C6/36 or Sf21 cells were transduced or infected with MOI = 10, harvested at various time-points, and intracellular viral DNA was quantified by qPCR. Amounts of viral DNA at all time-points were normalized against an internal gene control—GAPDH for Sf21 or Actin for C6/36—and further normalized against viral DNA amount after 2 h post-infection or post-transduction. Data represent the average ± SD of three biological replicates (*n* = 3).

Insect Sf21 cells are a natural host and permissive for baculovirus infection and DNA replication^29^. Therefore, we were curious to investigate whether baculovirus could replicate in mosquito C6/36 cells. To this end, we infected Sf21 or transduced C6/36 cells with vAB*sp*mC simultaneously. Time-course mCherry fluorescence images of C6/36 cells transduced with vAB*sp*mC at MOI = 10 are shown in Fig. 3a. Then, we examined baculovirus entry efficiency in both hosts at MOI = 10 and MOI = 50. Relative amounts of intracellular viral DNA at respective MOIs 2 h post-infection/transduction were found to be similar in both hosts, suggesting that baculovirus enters with similar efficiency into C6/36 and Sf21 cells (Fig. 3b). Next, we investigated the kinetics of intracellular viral DNA accumulation as a function of time and found a gradual increase of intracellular viral DNA from 12 h to 48 h post-infection in Sf21 cells, and saturation thereafter up to 96 h post-infection. In contrast, viral DNA gradually decreased as a function of time in C6/36 cells from 12 h to 96 h post-transduction, suggesting that baculovirus likely does not replicate in mosquito C6/36 cells, at least not replicate in a detectable level (Fig. 3c).

It is well known that baculovirus is replication-incompetent but capable of transducing into various mammalian cells, and the BacMam system is well established for popular applications in these cells^30^. We wished to compare baculovirus transduction efficiency between C6/36 (BacMos) and mammalian (BacMam) hosts. Mammalian cells are usually transduced with baculovirus at high MOIs of more than several hundred to several thousand^19^. We transduced C6/36 and commonly used mammalian Vero-E6^31^ and HEK-293T^32,33^ cells with MOIs of 1, 10 and 100, and then took mCherry fluorescence images at 48 h post-transduction (Supplementary Fig. 1a). Flow cytometry analysis showed that percentages of mCherry-positive cells increased with increasing viral dose for all three cell lines. The transduction efficiency was much higher in C6/36 cells compared to mammalian cell lines at all tested MOIs (Supplementary Fig. 1b), suggesting that mosquito C6/36 hosts are more permissive for baculovirus compared to mammalian hosts.

The most commonly used method to express foreign genes in mosquito hosts is the classical plasmid transfection approach. We compared transgene expression levels by baculovirus transduction versus plasmid DNA transfections. In order to do this, we transduced C6/36 cells with several doses of vAB*sp*mC (MOI = 1, 2 or 3) or used the plasmid pAB*sp*mC (10 kb) (DNA = 1 µg, 2 µg or 3 µg) originally inserted into the recombinant baculovirus for plasmid-based transfections (Supplementary Fig. 2a). Flow cytometry revealed that plasmid transfection efficiencies were around 15–20%, i.e., much lower than the baculovirus transduction efficiencies of around 60% at MOI = 1 (Supplementary Fig. 2b). Furthermore, relative transgene expression levels were significantly higher by baculovirus transduction than plasmid DNA transfection (Supplementary Fig. 2c).

### Functional analysis of various baculovirus-incorporated promoters and their efficiencies in C6/36 cells

**Figure 4:**
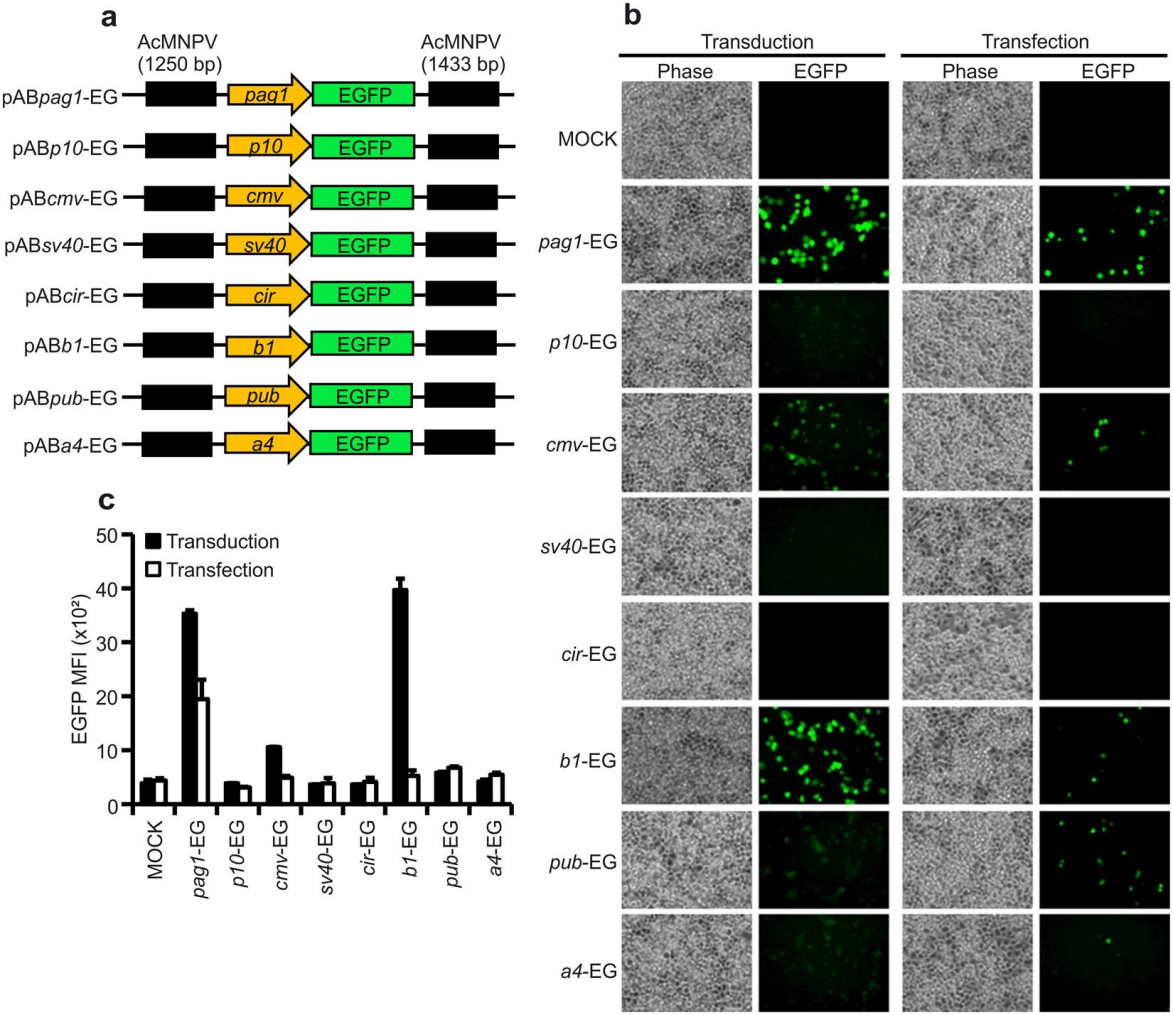
Efficiency of baculovirus-incorporated promoters in C6/36 cells. (**a**) Schematic representation of transfer vectors used to generate the recombinant baculoviruses expressing EGFP driven by several baculovirus, mammalian viral, and mosquito host promoters from the following genes: *pag1*, a HzNV-1 viral early expressing gene^23^; *p10*, baculovirus late gene^46^; *cmv*, cytomegalovirus^47^; *sv40*, simian virus 40^24^; *cir*, chimeric internal ribosome entry site (IRES) of RhPV virus and EV71 virus^48,49^; *b1*, cecropin b1 gene^50^; *pub*, polyubiquitin gene^42^ and *a4*, defensin a4 gene^51^. (**b**) Fluorescence images. C6/36 cells were transduced with recombinant viruses expressing EGFP at an MOI = 1 or transfected with 500 ng of the recombinant plasmid DNA constructs used to generate recombinant baculoviruses. The images were taken fluorescence microscopy at 48 h post-transduction. (**c**) Flow cytometry analysis of EGFP fluorescence driven by the promoters of interest. Recombinant baculovirus-transduced or plasmid DNA-transfected C6/36 cells were collected after 48 h and mean EGFP fluorescence intensities were measured by flow cytometry to determine promoter efficiency. Data represent the average ± SD of three biological replicates (*n* = 3).

Next, we tested the functionality and efficiency of various promoters by inserting their DNA sequences into the baculovirus genome. As reported earlier^34^, mosquito densovirus expression cassettes suffer from a size limitation for inserts. However, baculovirus does not have such size limitations so we constructed transfer vectors with different promoter sequences driving EGFP expression, as shown in Fig. 4a. After packaging of each transfer vector into baculovirus, C6/36 cells were transduced at MOI = 1 so that the majority of cells contained one or fewer viral genomic copies per cell. For comparison, C6/36 cells were also transfected with 500 ng plasmid of the same transfer vector so that each cell was transfected with an average of 1.15 × 10^5^ copies of plasmid DNA. EGFP images were taken at 48 h post-transduction (Fig. 4b).

We evaluated the strength of each promoter in C6/36 cells by quantifying mean fluorescence intensities of EGFP-positive cells by flow cytometry. We found *pag1* and *b1* promoters are consistently stronger among others, visually looked similar but the quantification data showed that *b1* driven EGFP expression was slightly better than *pag1* driven EGFP expression. In contrast, the *p10, sv40, cir, pub*, and *a4* promoters exhibited little or no fluorescence, and the *cmv* promoter only showed minimal activity. In our plasmid transfection system, *pag1* promoter showed better activity while the activity of other promoters remained basal or below the detection limit. Furthermore, under plasmid transfection, the *p10, sv40, cir*, and *a4* promoters exhibited similar results to baculovirus transduction (i.e., no fluorescence), and the *cmv* promoter only presented basal activity. However, the *pub* promoter functions better by plasmid transfection than baculovirus transduction. As expected, the baculovirus very late gene, *p10* promoter was not functional, further supporting that baculovirus is not a replicative virus in mosquito C6/36 cells (Fig. 4c).

We then introduced *b1* promoter-EGFP sequence into an established bi-cistronic baculovirus transfer vector^35^ containing 5′UTR internal ribosome entry site (IRES) of the *Rhopalosiphum padi virus* (RhPV) fused to EGFP. The *b1*-EGFP is reserved as a site for future foreign gene insertion in the BacMos to be expressed in mosquito hosts, and IRES-EGFP will serve as reporter protein in insect Sf21 host and facilitated the isolation of the recombinant AcMNPV. The resultant baculovirus, vBac*b1*EG-irEG showed significant enhancement in the EGFP expression compared to vAB*b1*EG virus in C6/36 cells (Supplementary Fig. 3a,b). Furthermore, we transduced vBac*b1*EG-irEG in other mosquito cell lines derived from *A. aegypti* (CCL-125) and *A. pseudoscutellaris* (AP-61) in addition to C6/36 cells. We found C6/36 has better transduction efficiency compared to CCL-125 and AP-61 cell lines transduced with different viral doses (Supplementary Fig. 3c,d) indicating baculovirus transduction is not cell-type specific. Our results demonstrate that the baculovirus-inserted *b1* promoter is the strongest among the tested promoters in mosquito C6/36 cells.

### Application of baculovirus mediated gene expression in mosquito C6/36 cell line

**Figure 5:**
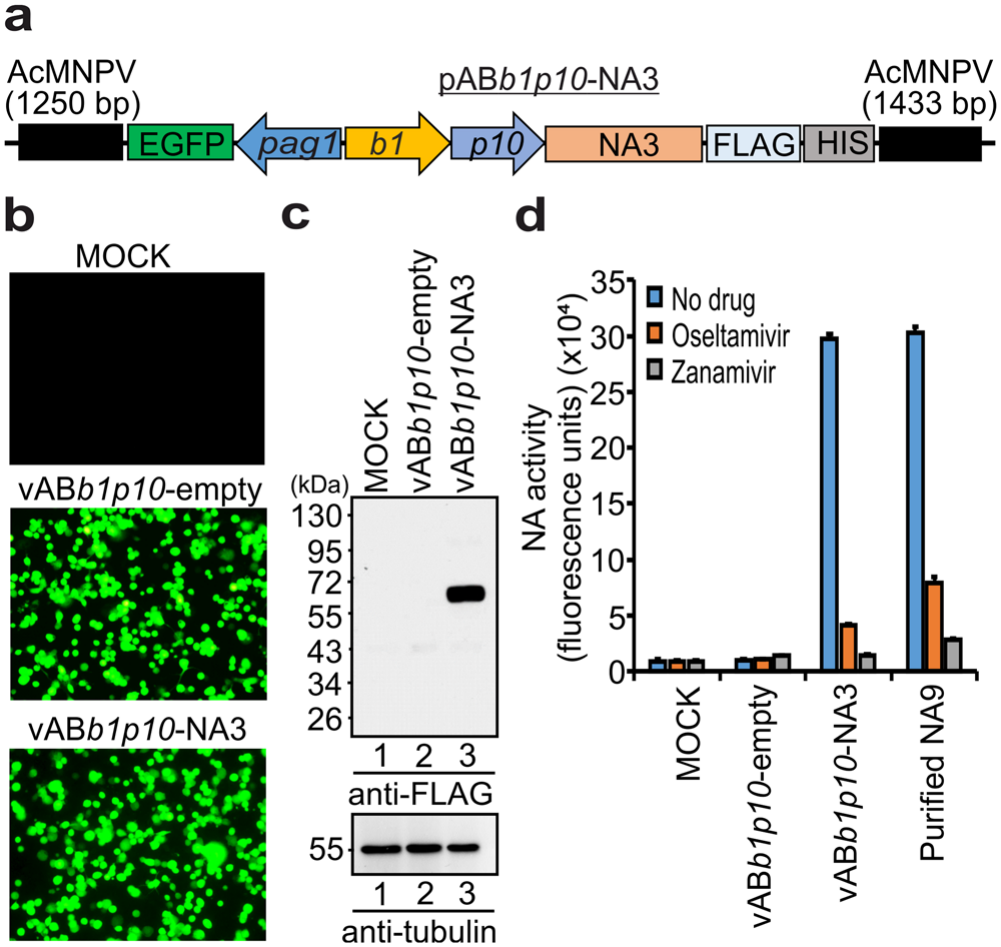
Enzymatic analysis of influenza virus NA expressed in mosquito C6/36 cells by recombinant baculovirus. (**a**) Schematic representation of a pAB*b1p10*-NA3 transfer vector for generating recombinant baculovirus. The transfer vector contains the EGFP reporter gene driven by the *pag1* promoter; NA (H5N3) gene under the control of dual host *b1* and *p10* promoters for mosquito C6/36 and insect Sf21 cells; FLAG and HIS tags were inserted at c-terminal; AcMNPV lateral ends flanking the expression cassette for homologous recombination. (**b**) C6/36 cells were transduced with vAB*b1p10*-empty (virus backbone without NA3) and vAB*b1p10*-NA3 baculovirus and the EGFP florescent pictures were taken at 48 h. (**c**) Western blot analysis. The cell lysates of MOCK, vAB*b1p10*-empty and vAB*b1p10*-NA3 were harvested at 48 h, analyzed by SDS-PAGE, followed by Western detection of NA3 protein in C6/36 cells. (**d**) Neuraminidase activity and Neuraminidase inhibition assays: The cell lysates of MOCK, vAB*b1p10*-empty and vAB*b1p10*-NA3 were mixed with or without (10 µM) NA inhibitors (Oseltamivir or Zanamivir) in the presence of 4-MUNANA substrate. Purified NA9 protein (H5N9 strain) was used as a positive control in the experiment. The experiment was performed three times and the data shown is the representative of one independent experiment done in triplicate. The error bars indicate the standard deviations.

Besides establishing the BacMos system using florescent reporter genes (mCherry and EGFP), we aimed to demonstrate the application of BacMos system by expressing the gene of interest which has functional assay that can be performed. In this study, we wished to express neuraminidase (NA) of influenza A virus H5N3 (NA3) and examine its enzymatic activity from the cell lysates. C6/36 cells were transduced with vAB*b1p10*-empty or vAB*b1p10*-NA3 baculovirus expressing *b1* promoter driven influenza NA3 (Fig. 5a). The EGFP florescence pictures revealed that these two viruses has similar transduction efficiency (Fig. 5b) and the cell lysates harvested at 48 h post-transduction were subjected to SDS-PAGE and Western blotting to examine the NA3 expression (Fig. 5c). The cell lysates were tested in florescence based assay that uses 4-methyl umbelliferone N-acetyl neuraminic acid (4-MUNANA) as a substrate to examine the neuraminidase activity (NA)^36^. We observed cell lysates from baculovirus mediated expression of NA3 showed a strong enzymatic activity in converting the substrate to the final products almost comparable to the activity resulted by the purified NA (H5N9 strain) protein, while the readings remained at basal level for the mock or vAB*b1p10*-empty virus transduced cell lysates. We also ensured the specificity of neuraminidase functions of NA by treating with well-known drug inhibitors such as Oseltamivir or Zanamivir (10 µM) which inhibited NA activity (Fig. 5d). This data demonstrates the application of baculovirus mediated gene expression for the desired genes in the mosquito model systems.

### Baculovirus-mediated gene expression in larvae and adults of various mosquito species

**Figure 6:**
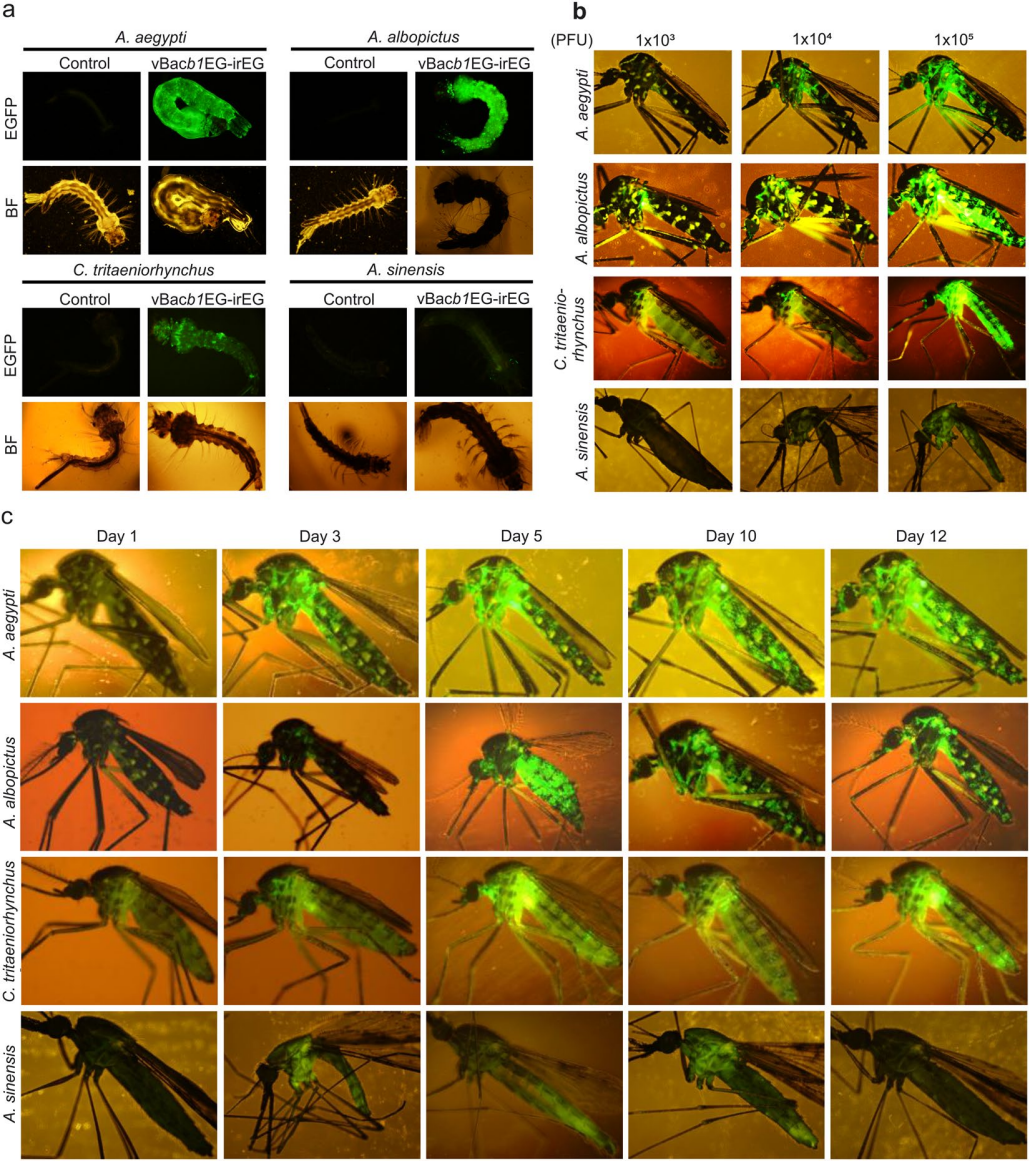
Analysis of *in vivo* baculovirus transduction of mosquitoes. (**a**) Transduction of baculovirus into mosquito larvae. Four different mosquito species (A. *aegypti, A. albopictus, C. tritaeniorhynchus*, and *A. sinensis*) were microinjected with 1 × 10^5^ PFU of vBac*b1*EG-irEG baculovirus. EGFP expression was visualized by fluorescence microscopy at 2 days post-transduction. (**b**) Dose-dependent expression of a baculovirus-mediated transgene in adult mosquitoes. Four different mosquito species were microinjected intra-thoracically with several doses (1 × 10^3^, 10^4^ or 10^5^ PFU) of vBac*b1*EG-irEG baculovirus. EGFP expression was visualized by fluorescence microscopy at 6 days post-transduction. (**c**) Transduction of baculovirus into adult mosquitoes. Four different mosquito species were microinjected intra-thoracically with 1 × 10^5^ PFU of vBac*b1*EG-irEG baculovirus. EGFP expression was visualized by fluorescence microscopy as a function of time over several days as shown.

Next, we examined the transduction ability of baculovirus into larvae of different mosquito species. *A. aegypti* (the yellow fever mosquito) and *A. albopictus* (Asian tiger mosquito or forest mosquito) are responsible for the transmission of various potentially fatal viruses in humans including dengue, chikungunya, zika, yellow fever, and Mayaro, as well as several filarial nematodes such as *Dirofilaria immitis* and other diseases. *Culex (Culex) tritaeniorhynchus* is the main vector of Japanese encephalitis and *Anopheles sinensis* transmits malaria, lymphatic filariasis^3^. We microinjected a dose of 1 × 10^5^ PFU recombinant vBac*b1*EG-irEG baculovirus into larvae of these four mosquito species and detected EGFP expression 2 days post-transduction. We observed strong EGFP expression in *A. aegypti* and *A. albopictus*, but weak expression in *C*. *tritaeniorhynchus* and *A*. *sinensis* (Fig. 6a).

**Figure 7:**
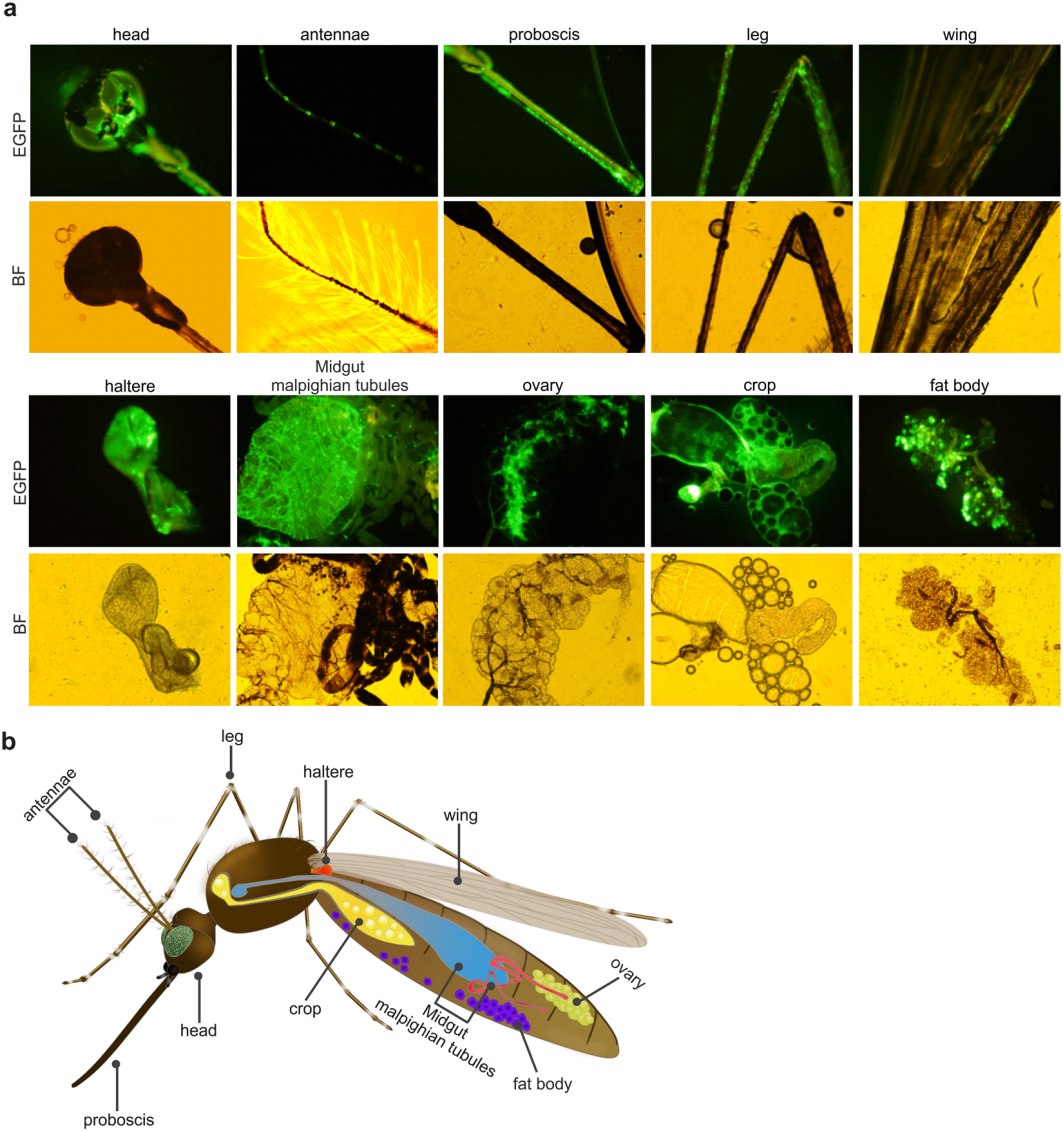
Tissue tropism of baculovirus in adult *A. aegypti*. (**a**) Dissection of baculovirus transduced adult mosquito. *A. aegypti* adult mosquitoes were microinjected intra-thoracically with 1 × 10^5^ PFU of vBac*b1*EG-irEG baculovirus and observed at 15 days post-transduction. EGFP fluorescence was observed in head, antennae, proboscis, leg, wing, haltere, midgut Malpighian tubules, ovary, crop, and fat body tissues of adult mosquitoes. EGFP: green fluorescence, and BF: bright field images. (**b**) Schematic representation of adult mosquito tissues. For simplicity, cartoon of adult mosquito showing various tissues representing respective images of (**a**) is shown.

We were also curious to examine the transduction ability of baculovirus into adult mosquitoes, so we assessed dose-dependent expression by intrathoracically microinjecting the same recombinant baculovirus (vBac*b1*EG-irEG). Interestingly, baculovirus-mediated EGFP expression in adult mosquitoes was higher with increasing viral dosages at 6 days post-microinjection. Again, we observed relatively weaker EGFP expression in *A. sinensis* compared to other three mosquito species (Fig. 6b). We also examined whether or not baculovirus replicates *in vivo* by performing qPCR on total genomic DNA isolated from transduced adult mosquitoes. We found that baculovirus does not replicate in adult mosquitoes (data not shown), corroborating our *in vitro* experiments. Next, we conducted a time-course experiment to repeatedly examine microinjected mosquitoes at regular time-points. EGFP expression was weak at 1 day post-injection, but increased thereafter throughout the experiment until 12 days post-injection (Fig. 6c). To determine the tissue tropism of baculovirus-mediated foreign gene expression, we dissected the transduced mosquitoes. EGFP expression was strongly observed in most of the tissues including head, proboscis, leg, haltere, midgut, ovary, crop, and fat body, but somewhat weak expression was observed in the Malpighian tubules, antennae, and wings (Fig. 7a). The schematic representation of the respective adult mosquito tissues are indicated (Fig. 7b). These results suggest that baculovirus can successfully transduce and express transgenes in the larvae and adults of the mosquito species tested here with little or no tissue barriers.

## Discussion

Mosquitoes are perhaps the most deadly animals for humans, transmitting disease to millions annually. Efficient gene delivery technologies are essential to advance studies of mosquito/pathogen interactions and mosquito biology. In this study, we have developed baculovirus as an efficient tool for gene delivery in mosquitoes.

Baculovirus has been widely used in various biotechnological applications such as protein production, gene transfer and also as a microbial insecticide^37,38^. Baculovirus was demonstrated possible for gene delivery into hepatocyte since the discovery of baculovirus as a gene delivery tool into non-host mammalian cell system^6,14^, after long year developments, it is now used in many mammalian cells for various fields of study and applications, including the transduction in many other cells, miRNA delivery, vaccine delivery, and for tissue engineering in mammals^21^. In this study, we show that baculovirus transduction by a dosage of MOI = 1 is enough to exhibit gene expression in about 55–60% of the cells (Fig. 1d) and it does not significantly affect the cellular proliferation rate but slightly a delay in the early time-points was observed. However, such slightly delay is quite nature due to baculovirus loading into the cells, and strong foreign protein expressions in these cells. Also, we observed that transduced cells were viable for up to 12 days at lower viral doses, with only mild effects being observed at MOI = 100. Our mCherry fluorescence images indicate that mCherry is persistently expressed up to 8 days post-transduction, and then slightly declined by 12 days post-transduction (Fig. 2f). We think this result is likely due to the transduced viral DNA being degraded or diluted upon cellular replication over time.

Although baculovirus is capable of transducing many mammalian cell lines, relatively high MOIs (usually 100–1000) are needed^19,39^. We found that baculovirus transduction efficiency was much higher in C6/36 cells compared to mammalian Vero-E6 and HEK-293T cells (Supplementary Fig. 1a,b). This is a significant finding because low MOIs decrease possible interference with cellular physiology by the viral transduction process. Further, we found that the efficiency of baculovirus transduction is greater than that of plasmid transfection and that protein expression was also much higher than that achieved through classical transfection approaches in C6/36 cells (Supplementary Fig. 2). Although we began our experiments with recombinant baculovirus (vAB*sp*mC, *pag1*-mCherry) containing a reasonably strong promoter, we discovered that the mosquito cecropin *b1* gene promoter is the strongest among our set of tested promoters through systematic comparison of transduction and transfection approaches in C6/36 cells (Fig. 4). The *pub* promoter consistently showed less fluorescence in the baculovirus transduction system but remained functional in the plasmid transfection system. We think this result could be due to the baculovirus-mediated cellular response suppressing *pub* promoter efficiency, or the promoter may not be well exposed in the context of viral genome. Similarly, this cellular response might have upregulated *b1* promoter activity in the baculovirus transduction system because cecropin *b1* is an immune-related gene; though the *a4* promoter is also from an immune-related gene, but its activity remained weak. Apart from C6/36 cells, the mosquito *b1* and *pub* promoters were also functional in insect Sf21 cells (data not shown), implying that these promoters could be functionally conserved to some extent in these evolutionarily divergent host cells.

In this study, we expressed NA of influenza H5N3 and analyzed its enzymatic activity from the baculovirus transduced C6/36 cell lysates as an example to demonstrate the application of BacMos system besides establishing using florescent reporter genes (Fig. 5). Baculovirus-mediated gene expression persisted for up to 12 days post-transduction, and the adult mosquitoes remained healthy and expressed transgenes in almost all tissues with little or no tissue barrier (Fig. 7a). Hence, it will be possible to study the function and regulation of the genes in different tissues using tissue specific promoters. It is noteworthy that baculovirus transduction could achieve maximum transgene expression at a low viral dose (1 × 10^5^ PFU) compared to the gene expression at a high viral dose (1 × 10^7^ PFU) by mosquito densoviruses (MDVs)^7^ and to plasmid injection methods^40^. In contrast to MDVs^7,41^, baculovirus likely does not exhibit species or tissue barriers, so construction of a single recombinant baculovirus is sufficient to study gene regulation in various mosquito species. Moreover, baculovirus will be possible in the future to deliver genes or DNA encoding small regulatory sequences into mosquito cells and organisms to study gene function, regulation, protein production and interactions, drug assays, virus/host interactions, and many other experiments in the cells or organisms with a flexibility whenever gene transduction is needed. BacMos system gives a flexibility to introduce or block a gene to analyze its function in a particular developmental stages from embryo to adults which is difficult to achieve by the conventional germ line engineering. Because, the deletion or over expression of many genes by germ line engineering could be lethal in the earlier developmental stages, therefore, limits the functional study of many such important genes in the later developmental stages. In these cases, baculovirus will be a novel alternative for the blocking or introducing of these genes transiently in different suitable developmental stages to study gene functions.

In summary, we have developed a BacMos system, a simple and efficient way to introduce foreign genes into the cells, larvae, and adults of many different mosquito genus. In contrast to transgenic mosquitoes, BacMos allows expression of a gene that may be lethal in early developmental stages but functions at other stages. Therefore, this robust BacMos system offers flexibility in the time of introduction, high level *in vivo* and *in vitro* gene expression, non-lytic transduction, broad host range, large DNA cargo capacity, and finally, with biosafety. Thus, our BacMos system exhibits considerable potential for the advancements of gene regulation studies in mosquitoes and associated viruses.

## Methods

### Cells and viruses

*A. albopictus* C6/36 and *A. aegypti* CCL-125 cell clones (ATCC, Taiwan) were maintained in RPMI-1640 medium (Invitrogen) supplemented with 10% fetal bovine serum (FBS), 2 mM L-glutamine, 0.1 mM non-essential amino acids, 1 mM sodium pyruvate, and 2% penicillin-streptomycin at 28 °C in a 5% CO_2_ humidified incubator. *A. pseudoscutellaris* AP-61 cell clone were maintained in L-15 medium (Leibovitz; GIBCO) + L-glutamine supplemented with 20% FBS and 2% penicillin-streptomycin at 28 °C in a 5% CO_2_ humidified incubator. The *Spodoptera frugiperda* IPLB-Sf21 (Sf21) cells (ThermoFisher Scientific) were grown at 26 °C in TC100 insect medium containing 10% FBS. HEK-293T and Vero-E6 cells (ATCC, Taiwan) were maintained in Dulbecco’s modified Eagle’s medium (DMEM; GIBCO) supplemented with 10% FBS and 2% penicillin-streptomycin at 37 °C in a 5% CO_2_ humidified incubator. All cell lines were routinely checked for mycoplasma every 2 months. The *Autographa californica* nucleopolyhedrovirus (AcMNPV) baculovirus (gene bank accession number NC_001623.1) genome (*flash*BACULTRA) was used to generate recombinant baculoviruses in this study. Viral frozen stocks in respective culture mediums were stored at −80 °C.

### Construction of transfer vectors and recombinant baculoviruses

The PCR amplified *TriEx* promoter from pTriEx™-3 vector (Novagen) was subcloned into pBacPAK8 (Clontech) replacing the *polyhedrin* promoter locus naming shortly as pAB transfer vector (p; plasmid, A; AcMNPV lateral ends, B; pBacPAK8 backbone vector) used for further modifications. In this plasmid, we have inserted mammalian *sv40* and *Hz*NV-1 *pag1* gene promoters upstream to the *TriEx* promoter. The PCR-amplified mCherry gene from pJET-mCherry vector was subcloned into downstream of the *pag1* promoter to generate the final transfer vector, named pAB*sp*mC. In this composite vector, mCherry expression is driven by the *sv40* and *pag1* promoters in the mammalian and insect systems, respectively. For promoter analysis, we constructed several transfer vectors as listed in Fig. 3a. Originally, the PCR amplified fragments of *hsp70* promoter and EGFP from different sources was subcloned into the *EcoR*V restriction site of pBluescript II SK (+) Phagemid (Agilent Technologies) to obtain pks/*hsp70*-EGFP plasmid. Then, the complete DNA fragment of *hsp70* promoter plus EGFP was subcloned in reverse orientation into the *EcoR*V restriction site of pBacPAK8 (Clontech), named as pAB*h*EG (*h*; *hsp70* promoter and EG; EGFP). The PCR-amplified various promoter regions were cloned upstream of EGFP into the pAB*h*EG plasmid replacing the *hsp70* promoter. The promoter regions of *pag1*, baculovirus early gene; *p10*, baculovirus late gene; *cmv*, cytomegalovirus; *sv40*, simian virus 40; *cir*, chimeric internal ribosome entry site (IRES) of RhPV virus and EV71 virus; as well as two mosquito genes cecropin b1 and defensin a4 (GenBank IDs: HQ285957.1, and HQ285959.1) both amplified by PCR from the *A. aegypti* genome; and the commercially-synthesized Polyubiquitin gene (GenBank ID: GU179018) promoter region (565 bp upstream of Pub gene)^42^ (named respectively as the pAB*pag1*EG, pAB*p10*EG, pAB*cmv*EG, pAB*sv40*EG, pAB*cir*EG, pAB*b1*EG, pAB*pub*EG, and pAB*a4*EG transfer vectors). The transfer vector pAB*sp*mC was modified in sequential manner to obtain the final pAB*b1p10*-NA3 plasmid used in Fig. 5; firstly the *sv40* promoter and mCherry gene was replaced by *pag1* promoter and EGFP gene upstream of *TriEx* promoter in the reverse orientation followed by replacing *cmv* and *T7* promoter by *b1* promoter upstream of *p10* promoter in the *TriEx* promoter. Next, the Neuraminidase (NA) of H5N3 (A/duck/Taiwan/a180/2015) influenza strain^43^, (named as NA3) was cloned in the multiple cloning site. At N-terminus of NA3, the signal peptide (SP) and cytoplasmic domain (CTD) of baculovirus envelope GP64 protein and FLAG and HIS tags at the C-terminus of NA3 were cloned respectively. These transfer vectors were constructed by following the standard protocols of the In-Fusion Cloning Kit (Clontech). The transfer vector pBac*b1*EG-irEG was constructed according to the traditional cloning methods. Briefly, a *Nco*I–*Xba*I EGFP fragment was subcloned into the *Nco*I and *Xba*I sites of pGL3 to create the plasmid, pGL3-EGFP. Both *Nhe*I–*Bgl*II promoter fragments of cecropin b1 were amplified and subcloned into pGL3-EGFP to create pGL3-*b1*EGFP. The restriction enzyme-digested region between *Nhe*I–*Not*I from pGL3-*b1*EGFP was subcloned into pBac-CHIKV-26S-Rhir-E^44^ replacing CHIKV-26S region. The large *Nhe*I-*Bgl*II fragment of pBac-*b1*EGFP-RhirE was fill-in and self-ligated to create the basic vector (shortly; pBac*b1*EG-irEG). All the above transfer vectors were verified by sequencing. Briefly, these transfer vectors were co-transfected with *flash*BACULTRA, a viral DNA of AcMNPV containing lateral fragments of 1250 bp and 1433 bp to facilitate homologous recombination, into Sf21 cells by using Cellfectin (Life Technologies). Homologous recombination occurs at the site of the polyhedron gene and produces a baculovirus lacking this gene. The resulting recombinant baculoviruses were isolated through end-point dilutions using florescent protein as the marker, amplifications in T75 flasks, and virus titers were determined by TCID_50_ analysis in Sf21 cells.

### Baculovirus transduction and infection

Mosquito C6/36 cells or mammalian cells were plated at a density of 4 × 10^5^ cells or 1 × 10^5^ cells (unless stated) per well in 24-well plates 1 day before transduction. The cells were washed with 1X DPBS and baculovirus was added at different MOIs as indicated in respective medium without any FBS or antibiotics. Plates were centrifuged at 2000 rpm for 32 min at room temperature (RT). Transduced mosquito or mammalian cells were incubated at 28 °C or RT, respectively, for 2 h. The plates were washed twice with 1X DPBS containing 0.1% trypsin to remove unattached viruses. Then complete 10%-FBS medium was added and cultured for various time-points as indicated. For baculovirus infection assays, Sf21 cells were plated at a density of 2 × 10^5^ cells per well in 24-well plates 1 day before infection and baculovirus was added at different MOIs. The plates were centrifuged at 2000 rpm for 32 min at RT and then incubated at 28 °C for various timeframes as indicated.

### Baculovirus neutralization assay

The vAB*sp*mC baculovirus (4 × 10^5^ PFU) at MOI = 1 was treated with 1 µg of neutralizing (AcV1, *Santa Cruz Biotechnology*) or non-neutralizing monoclonal antibodies (AcV5, *Santa Cruz Biotechnology*) against GP64 protein in a total volume of 300 µl with 1X DPBS and centrifuged at 300 rpm for 1 h at RT. The virus + antibody mixture was then added to C6/36 cells for 2 h and kept at 28 °C in a 5% CO_2_ humidified incubator. The virus + antibody mixture was then removed and washed three times with 1X DPBS containing 0.1% trypsin to remove unattached virus particles. Complete 10%-FBS medium was then added, and the cells were cultured for 2 days before examining mCherry fluorescence.

### Cell proliferation dye and cell viability analyses

C6/36 cells (4 × 10^5^) were seeded in 12-well culture dish, and the next day cells were transduced with recombinant vAB*sp*mC baculovirus at an MOI = 1, 10 and 100 for 2 hours. The unattached virus was removed, washed twice with 1X DPBS containing 0.1% trypsin, and 1 ml 10 µM cell proliferation dye (CPD) eFluor450 (eBioscience, catalog number: 65-0842) in 1X DPBS was added per well. Then, incubated at 28 °C in the dark for 10 min and the labeling was stopped by adding 3 ml of cold complete media keeping on ice for 5 min. Cells were washed 3 times with complete media and then cultured in complete media at 28 °C incubator. The experiment was performed in three biological replicates, three wells of cells were harvested immediately (time zero), followed by harvesting of samples 24, 48, 72 and 96 h. Cells were harvested by trypsinization, washed twice in 1X DPBS and prepared cells for flow cytometry analysis. For cell viability analysis, relative cell metabolic activity was determined by adding 10% v/v AlamarBlue to the cells and incubating for 4 h. Reductions in AlamarBlue concentrations were measured with a fluorescence reader (EnSpire, PerkinElmer) at an excitation wavelength of 560 nm and an emission wavelength of 590 nm.

### Plasmid DNA transfection

Mosquito C6/36 cells were plated at a density of 4 × 10^5^ cells per well in 24-well plates. The next day, various doses of recombinant plasmid DNA (1 µg, 2 µg and 3 µg in Supplementary Fig. 2 and 0.5 µg in Fig. 3) were mixed with respective amounts of transfection reagent (TransIT-Insect Transfection Reagent; Mirus) as per manufacturer recommendations and incubated for 30 min at RT, before the DNA + transfection reagent mixture was added to the cells. After 5 h in a 28 °C incubator, the cells were removed and washed with 1X DPBS, before 10%-FBS complete medium was added. The cells were then incubated at 28 °C for 2 days before being collected for flow cytometry analyses.

### Preparation of cells for flow cytometry analyses

Mosquito C6/36 cells were transduced with baculovirus in 24-well plates for various timeframes as shown in the figures. The medium was removed from the wells, cells were washed twice with 1X DPBS, and then treated with 100 µl of pre-warmed trypsin-EDTA for 5 min. Then 100 µl of RPMI-1640 medium (Invitrogen) was added to neutralize the trypsin-EDTA and cells were collected in 1 ml of 1X DPBS in 1.5 ml Eppendorf tubes. The cells were centrifuged at 5000 rpm for 5 min to remove the DMEM and the cell pellet was washed at least three times with 1X DPBS. Finally, cells were resuspended in 1 ml of fixing buffer (1% FBS + 1% formaldehyde diluted in 1X DPBS) and analyzed by flow cytometry. The numbers of mCherry- or EGFP-positive cells and the mean fluorescence intensities (MFI) were determined using flow cytometry and FlowJo software.

### Quantification of baculovirus DNA by real-time qPCR

Baculovirus-infected Sf21 or baculovirus-transduced C6/36 cells were extensively washed three-times with 1X DPBS containing 0.1% trypsin to remove free virus particles from cells. Total cellular DNA was extracted using the High Pure PCR Template Preparation Kit (Roche). The quantity and quality of DNA was checked using a NanoDrop ND-1000 spectrophotometer (Thermo Fisher Scientific). Real time-qPCR was performed in a 96-well plate, with each well containing 2 μl DNA (100 ng/µl), 0.6 μl 10 μM specific primers (final concentration, 300 nM), 6.8 μl H_2_O, and 10 μl 2X SYBR Green Master Mix (ABI). Samples were run in triplicate. Real-time PCR was conducted in the ABI 7500 FAST system for over 40 cycles with an annealing temperature of 60 °C. Assessment of the expression of each target gene was based on relative quantification (RQ) using the comparative critical threshold (CT) value method. The RQ of a specific gene was evaluated in each reaction by normalization to the CT obtained for the endogenous control genes (Actin gene in C6/36 cells and GAPDH in Sf21 cells). Three independent transfection experiments were conducted, and the data presented represent the results from one independent infection experiment. The primers for quantitative real-time PCR used in this study were as follows: primers for endogenous internal control: ACTIN forward (5′-CACGAACTGGGACGATATGG A-3′) and ACTIN reverse (5′- CGGTTAGCCTTCGGGTTCAG-3′); primers for baculovirus DNA amplification: IE-1 forward (5′-TTCGAATCCCTTGAGCAGC-3′) and IE-1 reverse (5′-TGCCGATGGTTGGTTCACA -3′); primers for endogenous internal control: GAPDH forward (5′-ATCAAGCAGAAGGTCAAGGAG-3′) and GAPDH reverse (5′-GCAGCAGCATCGAAGATAGA-3′).

### Western blot analysis

Mosquito C6/36 cells were transduced with vAB*b1p10*-empty (virus backbone) or vAB*b1p10*-NA3 at MOI = 10 in 24-well plates and cultured for 48 h. The culture medium was removed and cell lysate was harvested in 100 µl of 1X Radioimmunoprecipitation assay buffer (RIPA buffer, Thermoscientific, product#89901) and protein concentration was quantified by Coomassie Plus Bradford assay kit (Thermoscientific). 50 μg of total protein was mixed with 5X sample buffer and heated at 95 °C for 10 min, and proteins were resolved by 10% SDS-PAGE and subjected to Western detection. Western transfer was performed on nitrocellulose membranes. After blocking with 5% skim milk, the blot was incubated with mouse monoclonal anti-FLAG antibody (1:5,000; GeneTex) and rabbit polyclonal anti-Tubulin (1:5,000; GeneTex), followed by horseradish peroxidase (HRP)-conjugated mouse or goat anti-rabbit IgG (1:5,000). The blot was processed for detection with a commercial T-Pro Lumilong Plus Chemiluminescence Detection Kit (T-Pro Biotechnology, Cat. No: JT96-K004M).

### Neuraminidase activity (NA) and Neuraminidase inhibition (NI) assays

Neuraminidase activity (NA) and Neuraminidase inhibition (NI) assays was performed using 4-MUNANA assay^36^. 4-MUNANA assay is based on the cleavage of a fluorescent substrate, 2′-(4-methylumbelliferyl)-α-D-N-acetylneuraminic acid sodium salt hydrate (4-MUNANA; Sigma-Aldrich, Cat: M8639) by influenza NA; and the yield of free 4-methylumbelliferone (4-MU) fluorescent product is then quantitated based on its fluorescence intensity. In the NA assay, 5 µg of total cellular protein was mixed with 30 µl of 1 × 4-MUNANA substrate (final concentration of 100 µM) by pipetting in 96-well plate. In the NI assay, 10 µM of NA inhibitors (Oseltamivir or Zanamivir) were added to the cell lysates and incubated at 37 °C for 30 min before adding the 4-MUNANA substrate. Samples in 96-well plate were then incubated at 37 °C for 1 h to catalyze the 4-MUNANA substrate. The reaction was terminated by adding 150 µl of stop solution (0.14 M NaOH in 83% ethanol). The fluorescence due to release of 4-methylumbelliferone was read using an excitation wavelength of 365 nm and an emission wavelength of 450 nm.

### Mosquito rearing

The *A. aegypti* (Kaoshiung strain), *A. albopictus* (Chung-Ho strain), *C. tritaeniorhynchus* (Peitou strain), and *Anopheles sinensis* (Yunlin strain) were reared at 27 °C and at 80% humidity under a 12-h light/dark cycle. Larval stages were fed on pulverized fish food, and adults were provided with 5% sucrose *ad libitum*. Mosquitoes were maintained and performed experiments in full compliance with the Ethical Committee Guidelines and Regulations set by the National Defense Medical Center, Taipei 114, Taiwan, ROC.

### Mosquito virus intrathoracic inoculation

Frozen stocks of viruses were thawed at 37 °C, and four or five 10-fold serial dilutions of virus were made in RPMI1640 medium. Stocks of viruses with appropriate RPMI 1640 medium dilution were injected with 0.98 nl volume into 4–5 day post-eclosion female *A. aegypti, A. albopictus, C. tritaeniorhynchus*, and *A. sinensis* mosquitoes (starved for 24 h). Cold-anesthetized mosquitoes were intra-thoracically inoculated with virus according to the previous reported protocol^45^.

### Statistics

Data were analyzed by Student’s t-test and two tailed P value of <0.05 was considered significant.

### Accession codes

Nucleotide sequences have been deposited in GenBank under the following accession codes: pAB*pag1*-EG: MH290746; pAB*p10*-EG: MH290747; pAB*cmv*-EG: MH290748; pAB*sv40*-EG: MH290749; pAB*cir*-EG: MH290750; pAB*b1*-EG: MH290751; pAB*pub*-EG: MH290752; pAB*a4*-EG: MH290753; pAB*b1p10*-NA3: MH290754; pBac*b1*EG-irEG: MH290755.

## Electronic supplementary material

Supplementary Information

## Data Availability

The authors declare that the data supporting the findings of this study are available from the authors upon reasonable request.
