# Supplementary material for: Baculovirus as an efficient vector for gene delivery into mosquitoes
Source: Sci Rep. 2018 Dec 12;8:17778. doi: 10.1038/s41598-018-35463-8 (PMC6290771; doi:10.1038/s41598-018-35463-8)
Supplement: Supplementary file 1 — Supplementary Information [file 41598_2018_35463_MOESM1_ESM.pdf]

Baculovirus as an efficient vector for gene delivery into mosquitoes

Nenavath Gopal Naik[1], Yu-Wen Lo[1], Tzong-Yuan Wu[2,3*], Chang-Chi Lin[4,5*], Szu-Cheng Kuo[4,5*], Yu-Chan Chao[1,6,7*]

Supplementary Figures

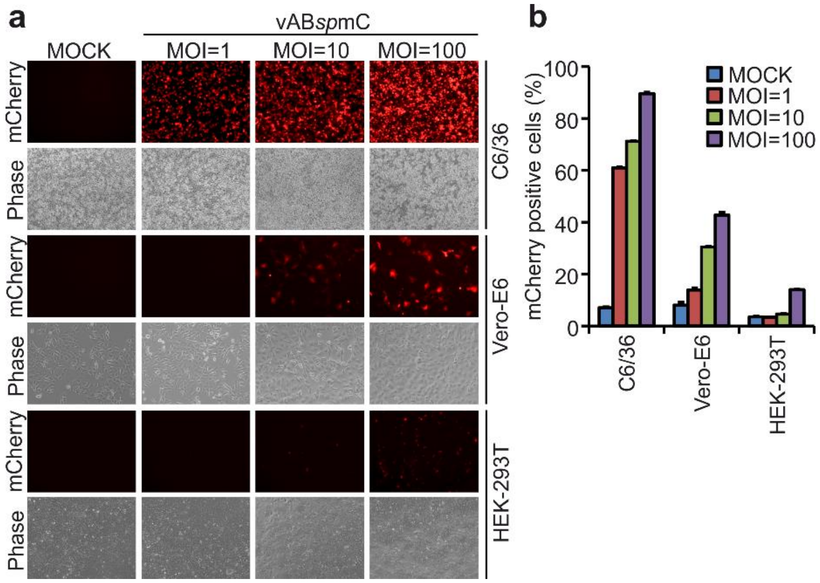

Supplementary Figure 1. Comparison of baculovirus transduction efficiency in mosquito C6/36 versus mammalian cell lines. (**a**) Representative mCherry fluorescence images of baculovirus transduction. Mosquito C6/36 or mammalian Vero-E6 and HEK-293T cells were transduced with vAB*sp*mC virus at MOI=1, 10 and 100. The mCherry reporter gene is driven by the *pag1* and *sv40* promoters in mosquito and mammalian cells, respectively. mCherry fluorescence images were captured at 2 days post-transduction by fluorescence microscopy. (**b**) Transduction efficiency. The transduced cells were collected for flow cytometry analyses to quantify the percentages of mCherry-positive cells (representing transduction efficiency). Data represent the average ± SD of three biological replicates (*n*=3).

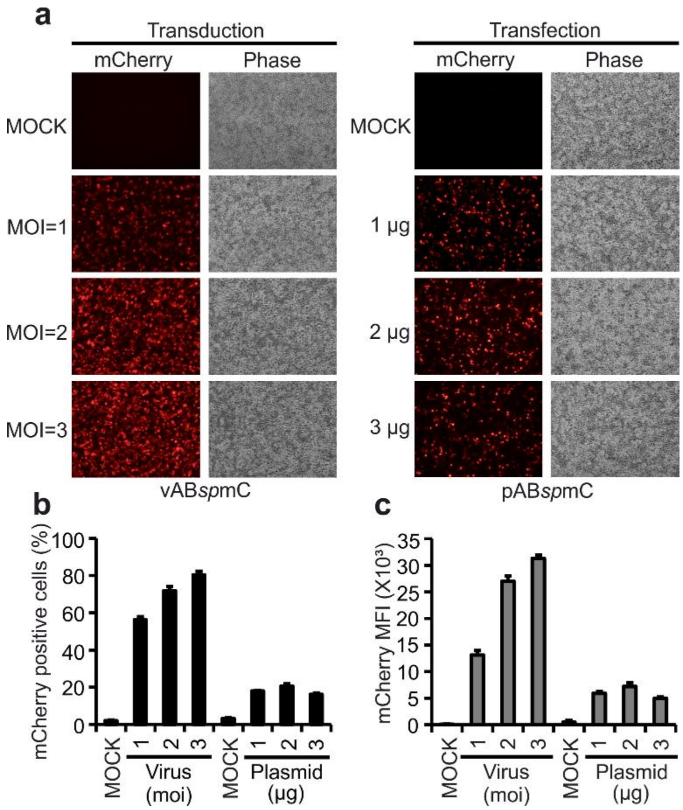

Supplementary Figure 2. Comparison of baculovirus transduction versus classical plasmid-based transfection in C6/36 cells. (**a**) The mCherry fluorescence images of baculovirus transduction and plasmid DNA transfection. Various concentrations of vAB*sp*mC were transduced into C6/36 cells. For plasmid transfection, pAB*sp*mC (10 kb), the original insert of the vABspmC recombinant baculovirus, was used. The mCherry fluorescence images were captured at 2 days post-transduction by fluorescence microscopy. (**b**) The mCherry-positive cells were quantified by flow cytometry to illustrate different transduction and transfection efficiencies. (**c**) The mean fluorescence intensity (MFI) of mCherry was quantified by flow cytometry. Data represent the average ± SD of three biological replicates (*n*=3).

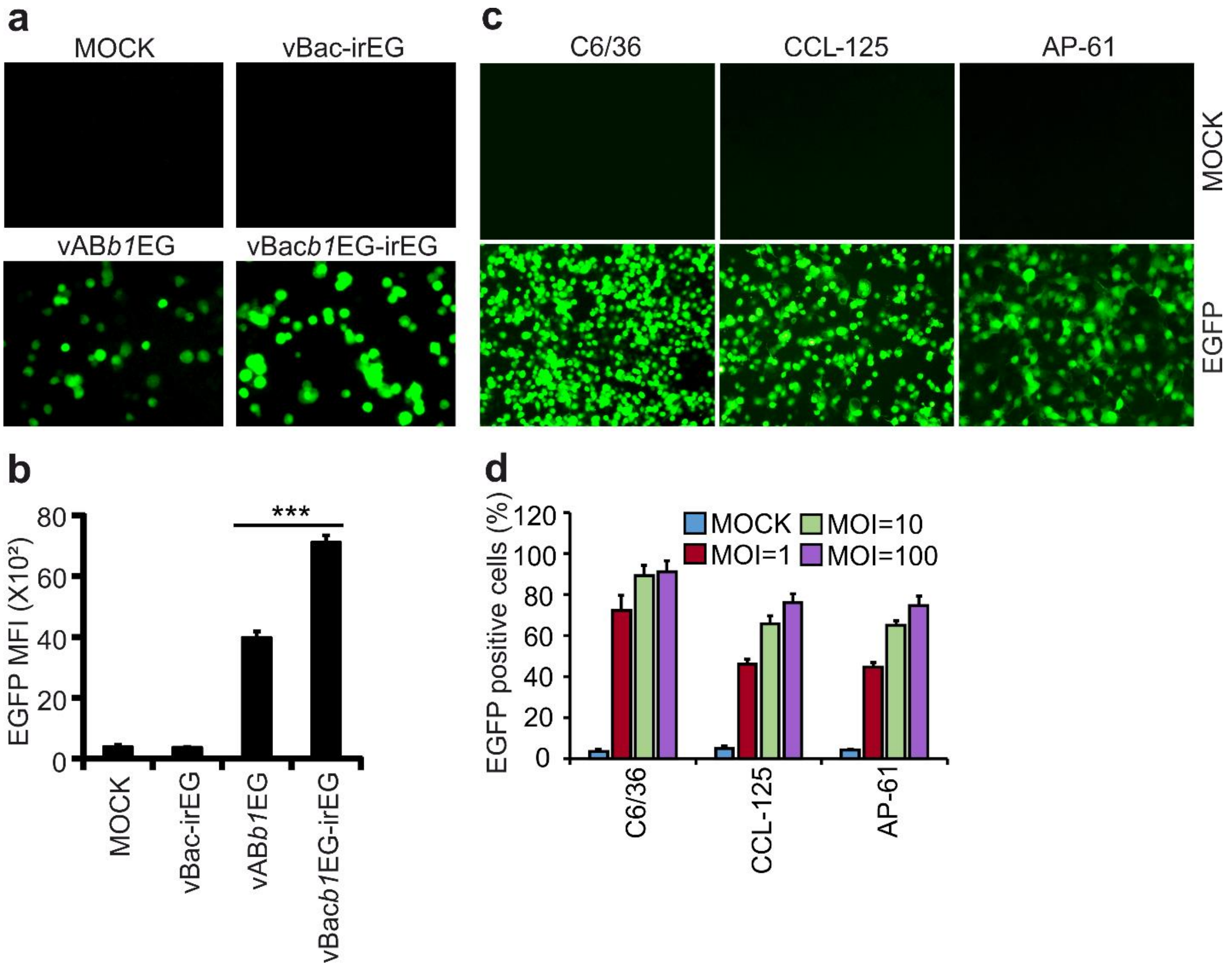

Supplementary Figure 3. Baculovirus transduction in mosquito cell lines is not cell-type dependent. (**a**) Analysis of EGFP expression in baculovirus inserted bi-cistronic expression cassette. Mosquito C6/36 cells were transduced with respective recombinant baculoviruses with MOI=1 and EGFP fluorescence images were captured at 2 days post-transduction by fluorescence microscopy. (**b**) The mean fluorescence intensity (MFI) of EGFP at 2 days post-transduction was quantified by flow cytometry. Data represent the average ± SD (standard deviation) of three biological replicates. Asterisks indicate statistically significant differences compared with the vAB*b1*EG virus, ****P*<0.0005 (two-tailed Student's *t* test). (**c**) Analysis of baculovirus transduction in different mosquito cell lines. *A. albopictus* C6/36, *A. aegypti* CCL-125 cells and *A. pseudoscutelians* were transduced with recombinant vBac*b1*EG-irEG, and EGFP fluorescence images were taken at 3 days post-transduction by fluorescence microscopy. (**d**) Transduction efficiency. C6/36, CCL-125 and AP-61 cells were transduced with vBac*b1*EG-irEG at MOI=1, 10 and 100. The number of EGFP-positive cells was quantified by flow cytometry at 2 days post-transduction to determine transduction efficiency. Data represent the average ± SD of three biological replicates (*n*=3).
